# Composite reconstruction of right ventricle and tricuspid valve for cardiac angiosarcoma

**DOI:** 10.1016/j.xjtc.2022.03.018

**Published:** 2022-04-18

**Authors:** Soichiro Kageyama, Takeki Ohashi, Yuji Kamikawa, Koichi Toda

**Affiliations:** a Department of Cardiovascular Surgery, Tokyo-Nishi Tokushukai Hospital, Tokyo, Japan; b Department of Cardiovascular Surgery, Nagoya Tokushukai General Hospital, Aichi, Japan; c Department of Cardiovascular Surgery, Osaka University Graduate School of Medicine, Osaka, Japan

**Figure:**
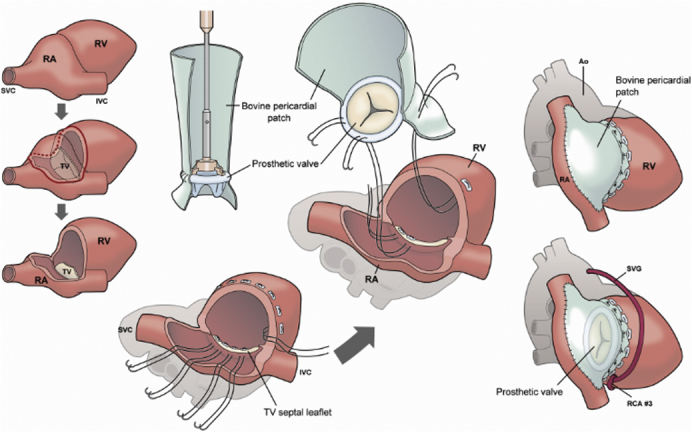
Composite reconstruction of the RA, RV, TV, and RCA for cardiac angiosarcoma.

**Central Message**

We reported extensive resection and reconstruction of the RA, RV, TV, and RCA using a novel composite graft for cardiac angiosarcoma.

Cardiac angiosarcoma is a rare malignancy characterized by aggressive local growth and early spread. Extensive invasion into the heart makes it difficult to perform surgery with curative intent because of the need for complicated heart reconstruction. We herein report a case of cardiac angiosarcoma recurrence that was subsequently treated with tumor resection and reconstruction of the right atrium (RA), right ventricle (RV), tricuspid valve (TV), and right coronary artery (RCA) using a novel composite graft.

## Case Report

**Figure 1:**
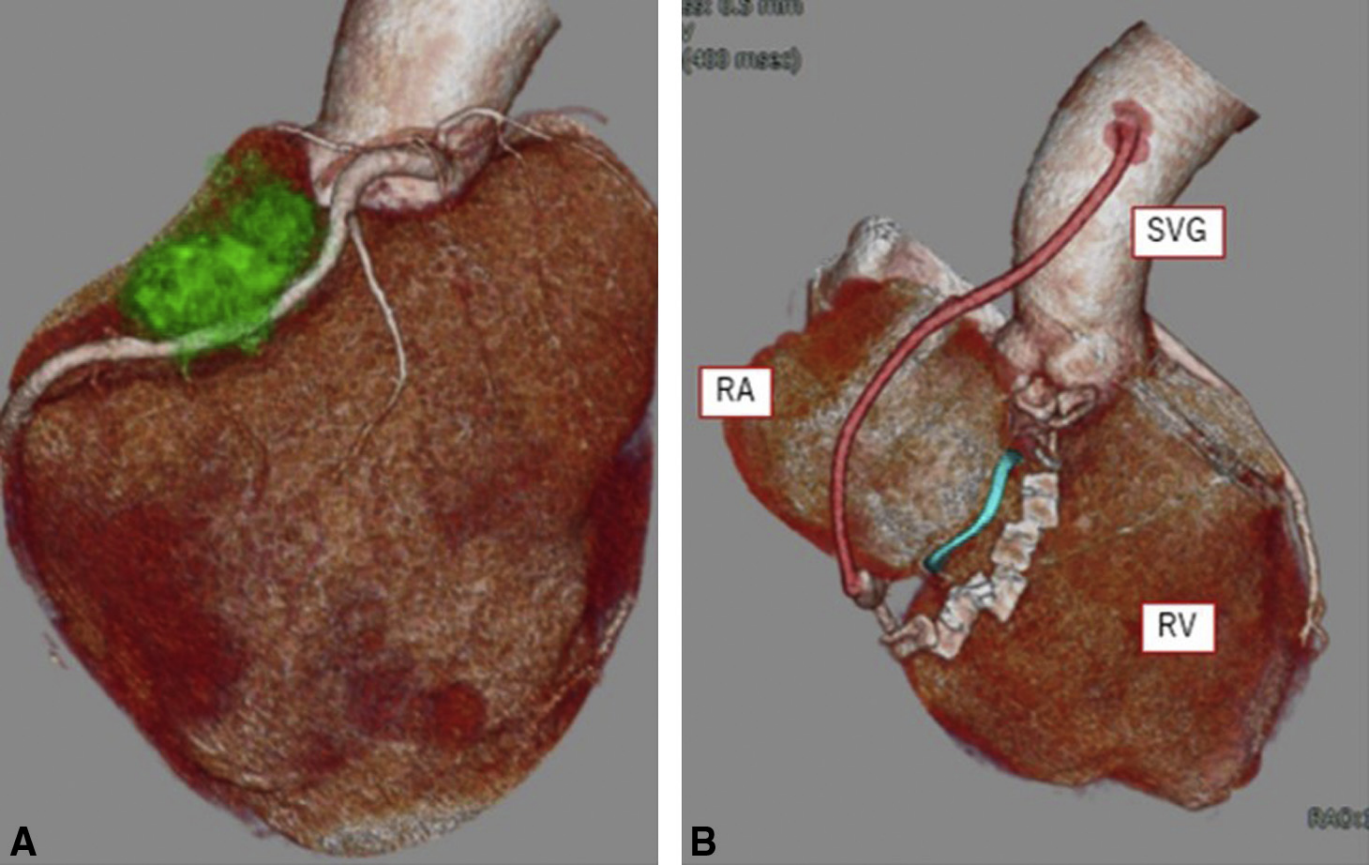
A, Cardiac contrast-enhanced CT scan showing recurrent tumor invading coronary artery and right ventricle. B, Cardiac contrast-enhanced CT scan after composite reconstruction. *SVG*, Saphenous vein graft; *RA*, right atrium; *RV*, right ventricular.

The patient was a 56-year-old male who initially underwent tumor resection with RA reconstruction at our facility. Written informed consent was obtained from the patient for publication of this case report; institutional review board approval was not required. A pathologic examination revealed an angiosarcoma, which was then completely resected with a negative margin. The positron emission tomography–computed tomography (PET-CT) scan findings obtained at 2 months after surgery did not demonstrate recurrence or metastasis. However, PET-CT scanning performed at 11 months postoperatively showed local recurrence in the RA wall, whereas cardiac contrast-enhanced CT scanning indicated that the recurrent tumor had invaded to the RV and even involved the RCA (Figure 1, *A*). Since PET-CT did not reveal metastasis, it was decided to perform another operation with curative intent at 12 months after the initial procedure.

## Operation

**Figure 2:**
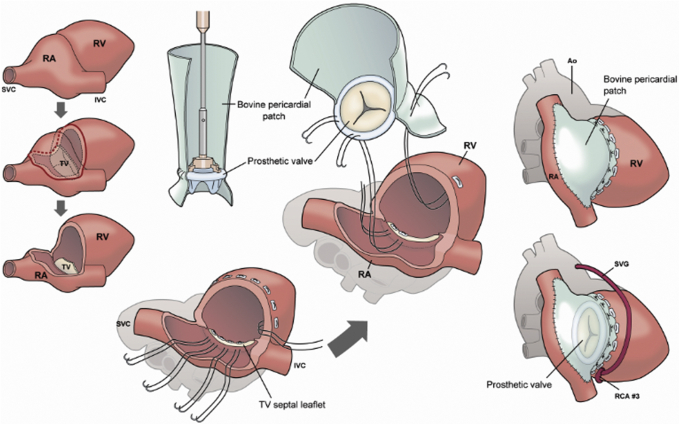
The tumor was excised with the right ventricular (*RV*) wall, anterior and posterior leaflets of the tricuspid valve (*TV*), right coronary artery (*RCA*), and right atrium (*RA*). A composite graft composed of a biological valve and bovine pericardial patch was prepared to form a skirt of approximately 3 cm on the RV side and approximately 10 cm on the RA side. Pledgetted 3-0 PROLENE sutures were placed in the tricuspid septal leaflet annulus and RV free wall, and then secured on the biological valve and the shorter skirt of the pericardial patch, respectively. Reconstruction of the RA was done using the longer skirt of the pericardial patch and RCA was reconstructed with a saphenous vein graft (*SVG*). *SVC*, Superior vena cava; *IVC*, inferior vena cava; *Ao*, ascending aorta.

Under a cardiopulmonary bypass, a saphenous vein graft was anastomosed to the RCA #3. The solid tumor was seen extending from the RA to RV wall and was excised along with the RV wall, anterior and posterior leaflets of the TV, RCA, and RA by making a fingerbreadth margin away from the tumor. A composite graft was prepared using a biological valve (29 mm, EPIC bioprosthetic valve; Abbott Inc) and bovine pericardial patch (Edwards Lifesciences). The pericardial patch was sewn over approximately two-thirds of the valve seat to form a skirt of approximately 3 cm on the RV side and approximately 10 cm on the RA side. After cardioplegic arrest, 16 pairs of pledgetted 3-0 PROLENE SH sutures (Ethicon) were placed in the tricuspid septal leaflet annulus and RV free wall and then secured on the biological valve and the shorter skirt of the pericardial patch, respectively. Reconstruction of the RA was performed using the longer skirt and RCA was reconstructed with saphenous vein graft (Figure 2, Video 1). Cardioplegic arrest time was 99 minutes.

The final pathologic diagnosis was cardiac angiosarcoma, and postoperative transesophageal echocardiography showed no residual tumor. Furthermore, transesophageal echocardiography indicated a left ventricular end-diastolic diameter of 31 mm, left ventricular ejection fraction of 57%, right ventricular end-diastolic diameter of 28 mm, and right ventricular fractional area change of 36.5%, the latter of which was as good as preoperative right ventricular fractional area change (38.1%). Postoperatively, central venous pressure was less than 10 mm Hg without inotropic support, and the patient did not show any symptom of right ventricular failure. However, after transfer to a referral hospital to receive adjuvant chemotherapy, his general condition rapidly deteriorated, and death occurred 2 months after the second surgery.

## Comment

Although attempts to repair the TV and RA after aggressive tumor resection have been reported, complete resection is difficult in most cases because of tumor invasion into adjacent cardiac structures.^1^ In the present case, a composite graft was constructed with a biological valve and bovine pericardium, before cardioplegic arrest. An advantage of the present approach is that it was possible to address defects of the RA, RV, and TV, which tend to change based on the amount of resection, by simply trimming the skirt of the composite graft.

A previous report noted the maximum resection limit of the RV free wall to be 30%,^2^ whereas others have reported patients who required a biventricular assist device after an extensive ventricular resection.^3^ In the present case, resection was performed up to 2 cm of the right ventricular free wall and then that part was reconstructed with a bovine pericardium skirt as part of a composite graft. Echocardiogram results at the time of discharge showed preserved right ventricle function. Although part of the SVC was resected and the septal sutures were passed through the tricuspid septal leaflet, the patient did not show sick sinus syndrome or atrioventricular block thereafter.

Unfortunately, the patient died, although the postoperative course was uneventful with only mild renal dysfunction (serum creatinine 1.7 mg/dL). An autopsy was not performed, and it remains unclear whether the sudden change in condition was due to sarcoma recurrence. A recent multicenter study indicated that neoadjuvant chemotherapy with radical surgery is safe and effective for cardiac sarcoma^4^; thus, earlier chemotherapy might have been necessary in the present case.

## Supplementary Data

Video 1 The video demonstrates the composite reconstruction technique used in the present case for recurrent cardiac angiosarcoma. (1) Under cardiopulmonary bypass, the SVG was anastomosed to the RCA. (2) The RA, RV wall, anterior and posterior leaflets of the TV, and RCA were resected with the tumor. (3) A composite graft composed of a biological valve and bovine pericardial patch was prepared. The pericardial patch was sewn over approximately two-thirds of the valve seat to form a skirt of approximately 3 cm on the RV side and approximately 10 cm on the RA side. (4) Sixteen pairs of pledgetted 3-0 PROLENE SH sutures were placed in the tricuspid septal leaflet annulus and RV free wall. Sutures on the septal annulus were secured to the valve seat of the biological valve, whereas those on the RV wall were secured to the shorter skirt of the pericardial patch. (5) After implantation of the composite graft, the RA was reconstructed using the longer skirt. (6) The proximal end of the SVG was anastomosed to the ascending aorta. *SVG*, Saphenous vein graft; *RCA*, right coronary artery; *RA*, right atrium; *RV*, right ventricle; *TV*, tricuspid valve. Video available at: https://www.jtcvs.org/article/S2666-2507(22)00231-0/fulltext.
