# Supplementary figures and images for: Composite reconstruction of right ventricle and tricuspid valve for cardiac angiosarcoma
Source: JTCVS Tech. 2022 Apr 18;13:67–9. doi: 10.1016/j.xjtc.2022.03.018 (PMC9196976; doi:10.1016/j.xjtc.2022.03.018)

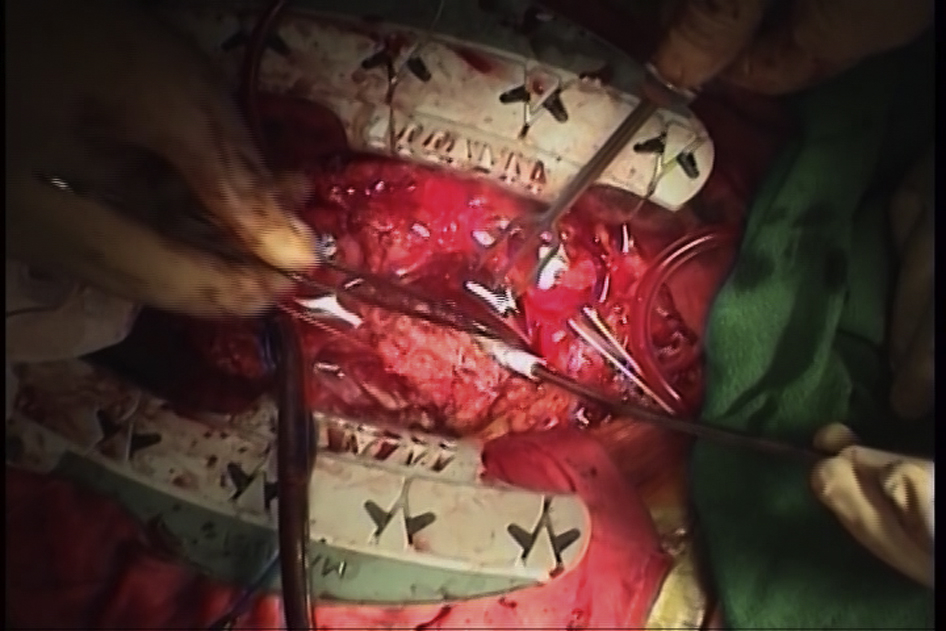

Supplement: Video 1 — The video demonstrates the composite reconstruction technique used in the present case for recurrent cardiac angiosarcoma. (1) Under cardiopulmonary bypass, the SVG was anastomosed to the RCA. (2) The RA, RV wall, anterior and posterior leaflets of the TV, and RCA were resected with the tumor. (3) A composite graft composed of a biological valve and bovine pericardial patch was prepared. The pericardial patch was sewn over approximately two-thirds of the valve seat to form a skirt of approximately 3 cm on the RV side and approximately 10 cm on the RA side. (4) Sixteen pairs of pledgetted 3-0 PROLENE SH sutures were placed in the tricuspid septal leaflet annulus and RV free wall. Sutures on the septal annulus were secured to the valve seat of the biological valve, whereas those on the RV wall were secured to the shorter skirt of the pericardial patch. (5) After implantation of the composite graft, the RA was reconstructed using the longer skirt. (6) The proximal end of the SVG was anastomosed to the ascending aorta. SVG, Saphenous vein graft; RCA, right coronary artery; RA, right atrium; RV, right ventricle; TV, tricuspid valve. Video available at: https://www.jtcvs.org/article/S2666-2507(22)00231-0/fulltext. [file fx2.jpg]
